# Role of Cysteine Residues in the Carboxyl-Terminus of the Follicle-Stimulating Hormone Receptor in Intracellular Traffic and Postendocytic Processing

**DOI:** 10.3389/fcell.2016.00076

**Published:** 2016-07-20

**Authors:** Brenda Melo-Nava, Patricia Casas-González, Marco A. Pérez-Solís, Jean Castillo-Badillo, José L. Maravillas-Montero, Eduardo Jardón-Valadez, Teresa Zariñán, Arturo Aguilar-Rojas, Nathalie Gallay, Eric Reiter, Alfredo Ulloa-Aguirre

**Affiliations:** ^1^Research Unit in Reproductive Medicine, Unidad Medica de Alta Especialidad Hospital de Ginecobstetricia “Luis Castelazo Ayala”, Instituto Mexicano del Seguro SocialMexico City, Mexico; ^2^Research Support Network, Universidad Nacional Autónoma de México and Instituto Nacional de Ciencias Médicas y Nutrición “Salvador Zubirán”Mexico City, Mexico; ^3^Department of Earth Resources, Universidad Autónoma MetropolitanaLerma, Mexico; ^4^BIOS Group, UMR85, Unité Physiologie de la Reproduction et des Comportements, Centre National de la Recherche Scientifique, Institut National de la Recherche Agronomique, UMR7247, Université François RabelaisTours, France

**Keywords:** follicle-stimulating hormone, follitropin, follicle-stimulating hormone receptor, palmitoylation, internalization, recycling

## Abstract

Posttranslational modifications occurring during the biosynthesis of G protein-coupled receptors include glycosylation and palmitoylation at conserved cysteine residues located in the carboxyl-terminus of the receptor. In a number of these receptors, these modifications play an important role in receptor function and particularly, in intracellular trafficking. In the present study, the three cysteine residues present in the carboxyl-terminus of the human FSHR were replaced with glycine by site-directed mutagenesis. Wild-type and mutant (Cys627/629/655Gly) FSHRs were then transiently expressed in HEK-293 cells and analyzed for cell-surface plasma membrane expression, agonist-stimulated signaling and internalization, and postendocytic processing in the absence and presence of lysosome and/or proteasome inhibitors. Compared with the wild-type FSHR, the triple mutant FSHR exhibited ~70% reduction in plasma membrane expression as well as a profound attenuation in agonist-stimulated cAMP production and ERK1/2 phosphorylation. Incubation of HEK-293 cells expressing the wild-type FSHR with 2-bromopalmitate (palmitoylation inhibitor) for 6 h, decreased plasma membrane expression of the receptor by ~30%. The internalization kinetics and β-arrestin 1 and 2 recruitment were similar between the wild-type and triple mutant FSHR as disclosed by assays performed in non-equilibrium binding conditions and by confocal microscopy. Cells expressing the mutant FSHR recycled the internalized FSHR back to the plasma membrane less efficiently than those expressing the wild-type FSHR, an effect that was counteracted by proteasome but not by lysosome inhibition. These results indicate that replacement of the cysteine residues present in the carboxyl-terminus of the FSHR, impairs receptor trafficking from the endoplasmic reticulum/Golgi apparatus to the plasma membrane and its recycling from endosomes back to the cell surface following agonist-induced internalization. Since in the FSHR these cysteine residues are S-palmitoylated, the data presented emphasize on this posttranslational modification as an important factor for both upward and downward trafficking of this receptor.

## Introduction

Follicle-stimulating hormone (FSH) or follitropin is a glycoprotein hormone synthesized by the pituitary gland that plays an essential role in the regulation of gonadal function. Its cognate receptor (FSHR) belongs to the G protein-coupled receptor (GPCR) superfamily and is mainly expressed in the granulosa cells of the ovary and in the Sertoli cells of the seminiferous tubule (Dias et al., 2002). Upon agonist binding, the activated FSHR stimulates distinct intracellular signaling cascades, mainly that involving the canonical Gαs/cAMP/PKA pathway (Richards and Pangas, 2010; Ulloa-Aguirre et al., 2011). As with other GPCRs, the FSHR undergoes agonist-stimulated desensitization and internalization, a process that involves phosphorylation by GPCR kinases and recruitment of β-arrestin 1 and 2, which link the receptor-agonist complex to components of the endocytic machinery (Krishnamurthy et al., 2003a,b; Ulloa-Aguirre et al., 2011). Although most of the FSH/FSHR complex accumulated in endosomes is subsequently recycled back to the cell surface plasma membrane (PM), a fraction of this complex is targeted for degradation by the proteasomal/lysosomal machinery (Krishnamurthy et al., 2003a).

Posttranslational modifications occurring during the biosynthesis of the FSHR include glycosylation and palmitoylation at conserved cysteine residues located in the carboxyl-terminus (Ctail) of the receptor (Davis et al., 1995; Ulloa-Aguirre et al., 2013). Among several functions, S-acylation with palmitic acid is required in many GPCRs, including the glycoprotein hormone receptors [FSHR, lutropin receptor (LHR), and thyrotropin receptor (TSHR)], for efficient intracellular trafficking and for anchoring the carboxyl-terminal domain of the receptor protein to the PM (Tanaka et al., 1998; Munshi et al., 2001, 2005; Qanbar and Bouvier, 2003; Linder and Deschenes, 2007; Uribe et al., 2008). Employing site-directed mutagenesis, we previously documented that the human FSHR is palmitoylated in its Ctail at two conserved cysteine residues (Cys629 and Cys655) and one nonconserved residue (Cys627), that is, at all cysteine residues regardless of their location in this domain (Uribe et al., 2008). While palmitoylation at either Cys627 or Cys655 is not essential for FSHR PM expression, S-acylation at Cys629 is extremely important, as replacement of this residue with alanine dramatically reduced expression of the mature receptor by 40–70% (Uribe et al., 2008). In contrast to the LHR, in which palmitoylation at conserved cysteine residues is determinant for agonist-stimulated internalization and postendocytic processing (Munshi et al., 2005), there is no information on the role of this posttranslational modification on the downward trafficking of the FSHR.

We here performed a series of experiments to examine the intracellular traffic of a triple human FSHR mutant with all Ctail cysteine residues mutated with glycine (FSHRC627/629/655G), with special focus on the postendocytic processing of the receptor. Gly instead of Ala was chosen to replace the Ctail Cys residues of the receptor to allow a better comparison of the downward trafficking parameters analyzed with those previously reported for the Ctail Cys → Gly mutant of the human LHR (Munshi et al., 2001, 2005). We found that the functional behavior (PM expression, agonist-stimulated signaling and internalization) and conformational changes of the triple mutant FSHRC627/629/655G were similar to those exhibited by the Cys → Ala mutant (Uribe et al., 2008). As a new information, we demonstrated that replacement of Cys residues at the Ctail of the FSHR, led to impaired recycling to the cell surface plasma membrane after agonist-stimulated internalization, an effect that was partially counteracted by inhibiting proteasome function. These data indicate that palmitoylation does not influence FSHR internalization but is important for its postendocytic processing and recycling back to the PM after exposure to agonist.

## Materials and methods

### Construction of the FSHRC627/629/655G

The plasmid designed for the expression of FSHRC627/629/655G was obtained after three consecutive site-directed mutagenesis reactions on the wild-type (WT) human FSHR cDNA (GeneBank accession no. S59900) cloned into the mammalian expression vector pSG-5 (Agilent Technologies, Santa Clara, CA, USA). Site-directed mutagenesis was performed employing the QuickChange II Site-Directed Mutagenesis kit (Agilent), following the manufacturer instructions. The sequence of mutagenic complementary oligonucleotides (Invitrogen, Carlsbad, CA, USA) used for the replacement of cysteine for glycine are shown in Table 1. Mutations at the target residues and the fidelity of the remaining nucleotide sequence were verified by automatic DNA sequencing. Large-scale plasmid DNAs for transfection were prepared using an Endofree maxiprep kit (Qiagen, Valencia, CA, USA).

**Table 1:** **Sequence of mutagenic complementary oligonucleotides employed to construct the triple Cys627/629/655Gly human FSHR mutant**.

**Residue**	**Oligonucleotides**
Cys627Gly	Forward: 5′-CTG CTG AGC AAG GGT GGC TGC TAT G-3′
	Reverse: 5′- C ATA GCA GCC ACC CTT GCT CAG CAG-3′
Cys 629Gly	Forward: 5′-G AGC AAG GGT GGC GGC TAT GAA ATG C-3′
	Reverse: 5′-G CAT TTC ATA GCC GCC ACC CTT GCT C-3′
Cys 655/Gly	Forward: 5′-AGG AAT GGC CAC GGC TCT TCA GCT C-3′
	Reverse: 5′-G AGC TGA ACA GCC GTG GCC ATT CCT-3′

### Computational modeling and molecular dynamics simulations of WT and triple mutant FSHRs

The FSHR model was generated by means of the GPCR-I-TASSER protocol (Zhang et al., 2015). The model with the highest C-score was the initial structure in a molecular dynamics (MD) set up. A preliminary validation of the FSHR model consisted of a comparison against a second model using the sequence Leu241 to Asn678 to test consistency of the predicted model. Upon fitting the backbone atoms from Leu348 to Gly628, which includes the transmembrane helices and interhelical loops, a root mean square deviation (RMSD) of 1.87 Å was calculated. All cysteines were predicted in the same context in both structures. Another preliminary validation was the transmembrane domains (TMD) prediction along the FSHR sequence, using the physicochemical and translocon hydropathy scales in the MPex server (Snider et al., 2009). From the comparison of the predicted TMD and the FSHR model, we concluded that the hydrophobicity of the FSHR model was suited for a relaxation in a lipid bilayer environment. Thus, the FSHR model was further refined using MD simulation for the protein Asp300 to Asn678, which contained a fragment of the amino-terminus, the transmembrane helices, and the complete Ctail. The FSHR model was embedded in a hydrated lipid bilayer of 1-stearoyl-2-docosahexaenoyl-sn-glycero-3-phosphocholine (SDPC) molecules. In order to remove any repulsive contact in the initial configuration, 3000 steps of energy minimization using the conjugate gradient algorithm were executed. After minimization, positional constraints were defined on the heavy atoms of the protein. Starting from a force constant of 25 kcal mol^−1^ Å^−1^, constraints were slowly removed in 200 ps steps with force constants of 15, 10, 5, 3, 2, and 1 kcal mol^−1^ Å^−1^. Simulations were extended during 20 ns for the FSHR model without any constrain at 300 K and 1 bar (NPT ensemble). The relaxed WT FSHR structure was also used to generate two mutants by replacing Cys627, Cys629, and Cys655 with glycine (Gly^3^ mutant) or alanine (Ala^3^ mutant). In all the MD simulations, the FSHR model included only the fragment Asp300 to Asn678, which contained an amino-terminus shorter than native FSHR, the seven transmembrane helices, and the complete Ctail. Trajectories of 20 to 24 ns long were generated for each mutant. According to available crystallographic data on GPCR (Murakami and Kouyama, 2008; Murakami Kouyama and Kouyama, 2015), water molecules in the interhelical region stabilize hydration of side chains and form hydrogen bond chains, which may play a role for the conformational changes toward the active state. Hence, water molecules in the interhelical region were preserved from a previous set up of squid rhodopsin in a SDPC lipid bilayer (Jardon-Valadez et al., 2010).

### Cell culture and transfection

Human embryonic kidney (HEK)-293 cells (ATTC, Manassas, VA, USA) in high-glucose DMEM supplemented with 5% fetal calf serum (FCS) and antibiotics were maintained in an humidified atmosphere of 5% CO_2_ at 37°C. Cells grown to 70–80% confluency in 10 cm diameter dishes were replated on 60 mm diameter dishes and cultured for 24 h at 37°C. Subconfluent cells (~750 × 10^3^ cells/dish) were transfected with 4 μg WT, mutant FSHR cDNAs or empty vector by liposome-mediated endocytosis in OPTIMEM (Life Technologies, Grand Island, NY). Transfected cells were then incubated for an additional 24 h before replating in 24-well-plates for subsequent experiments or processed for immunoblotting. Co-transfections of WT and mutant FSHR with cDNA constructs of β-arrestins [β-arrestin 1 and 2, or a dominant-negative form of β-arrestins (β-arrestin 319–418), Krupnick et al., 1997] were performed employing 3 μg of WT or mutant FSHR cDNA plus either 1 μg β-arrestin 1, β-arrestin 2, β-arrestin 319–418, or empty vector. In some experiments, cells expressing the WT FSHR were exposed to 50 μM 2-bromopalmitate (an irreversible S-palmitoylation inhibitor) (Sigma Aldrich, St. Louis, MO, USA) for 6 h, time during which cell viability was not affected as assessed by the yellow tetrazolium salt XTT assay (Roche Applied Science, Mannheim, Germany), before the binding assay or preparation of protein extracts for SDS-PAGE.

### SDS-PAGE and immunoblotting

SDS-PAGE (7.5%) and Western blotting of whole cell lysates from cells expressing the WT or C627/629/655G cDNAs were performed as described previously (Ulloa-Aguirre et al., 2013) employing the primary anti-human FSHR antibody mAb106.105 (Lindau-Shepard et al., 2001) and the secondary anti-mouse IgG horseradish peroxidase conjugate (Biosource International, Armadillo, CA, USA). Signal was developed using the Pierce ECL Western Blotting detection kit (Rockford, IL, USA). Equal protein loading was verified in a reprobed membrane with a 1:10000 anti-glyceraldehyde-3-phosphate dehidrogenase (GAPDH) antibody (Sigma) and 1:15000 goat-anti-mouse IgG conjugated with horseradish peroxidase (Biosource). In some experiments, protein extracts (20–40 μg protein) were incubated with 2000 units of Endo H (dissolved in 50 mm sodium citrate pH 5.5) (New England Biolabs, Beverly, MA, USA) at 37°C for 16 h and the reaction was stopped by the addition of Laemmli sample buffer before processing for 7.5% SDS-PAGE and immunoblotting.

### Receptor binding assay

Binding of ^125^I-FSH to the FSHR transiently expressed in HEK-293 cells was assessed as previously described (Ulloa-Aguirre et al., 2013). Cells cultured in 60 mM dishes were transfected with the FSHR cDNAs and 24 h after transfection the cells were replated in 24-well-plates. Forty-eight hours after transfection, the medium was removed, replaced with fresh medium, and allowed to continue incubation at 37°C for 1 additional hour. After the preincubation period, the medium was removed and serum-free DMEM containing 20 ng/ml ^125^I-FSH (specific activity ~28 μCi/μg protein) was added to each well in the absence or presence of ~1 μg/ml recombinant FSH (to assess for non-specific binding). Hormone was allowed to bind for 1 h at 37°C and thereafter the plates were placed on ice and washed twice with ice-cold PBS. Cell-surface ^125^I-FSH was eluted with ice-cold 50 mM glycine/100 mM NaCl, pH 3.0 (elution buffer), for 10 min on ice and the eluate was removed to a glass tube and counted.

### Measurement of cAMP production

Forty-eight hours after transfection, the medium was removed and the cells in 24-well-dishes were washed twice with DMEM-5% FCS and then stimulated with increasing doses of human recombinant FSH (Merck-Serono, Mexico D.F., Mexico) in the presence of 0.125 mM 3-isobutyl-methyl-xanthine (Sigma). At the end of the incubation period (18 h), the medium was removed and total (extracellular plus intracellular) cAMP accumulation was measured by radioimmunoassay (Zambrano et al., 1996).

### Internalization of the FSHR under nonequilibrium binding conditions

Internalization of the FSHR was performed as described previously (Ulloa-Aguirre et al., 2013). Briefly, HEK-293 cells transfected with the WT or mutant FSHR cDNAs or cotransfected with WT or mutant FSHR cDNAs and either β-arrestin 1, β-arrestin 2, β-arrestin 319-418, or empty vector were seeded into 24-well-plates pretreated with poly-D-lysine (50 μg/ml) (Sigma) at 1.5 × 10^5^ cells/ml/well, and incubated until subconfluency. After a 60 min preincubation period in serum-free DMEM, cells were exposed to 20 ng/ml ^125^I-FSH in the presence or absence of 1 μg unlabeled recombinant FSH for 0 to 90 min or for 2 h at 37°C. At each time point cells were placed on ice, washed with PBS, and incubated on ice in elution buffer for 20 min to recover cell-surface ^125^I-FSH. After the elute was removed for counting, cells were washed with PBS and solubilized in 2N NaOH for 1 h at room temperature to allow detection of cell-associated counts per minute. The FSHR internalization rate is expressed as the internalized (cell-associated)/surface ^125^I-FSH ratio.

### Confocal microscopy

Confocal microscopy was conducted to localize cell surface and internalized FSHRs before and after FSH stimulation. Transfected HEK-293 cells were cultured in Histogrip (Invitrogen)-coated coverslips and exposed to 100 ng/ml FSH for 2 h at 37°C. Cells were then fixed with 2% paraformaldehyde in PBS at 37°C for 30 min and bleached with 0.1 M glycine for 5 min. Cells were permeabilized with 0.05% Triton X-100 in PBS for 10 min at 4°C and the slides were incubated in 1% bovine serum albumin dissolved in PBS for 1 h at room temperature to block nonspecific sites. Cells were then incubated with the primary antibody Mab106.105 (1:200) for 12 h at 4°C and then with 1:200 FITC-conjugated anti-mouse IgG antibody (Millipore, Temecula CA, USA) for 1 h at room temperature. Slides were mounted in ProLong antifade reagent (Invitrogen) and imaged on an Olympus FlowView FV10i confocal laser scanning microscope (Olympus, Tokyo, Japan). The FV10-ASW sofware (Olympus) was employed for imagen processing and analysis. The FSHR fluorescence signal intensity across the plane of the cells was analyzed using ImageJ 3D and Plot Profile analysis (National Institutes of Health, Bethesda, MD, USA; https://imagej.nih.gov/ij/), which displays the intensity of pixels over the whole image surface or along a line as a 2-dimensional graph. Lines were traced over the images obtained by confocal microscopy to obtain the intensity values using the Plot Profile plug-in. The values so obtained were plotted using the Prism 6 software (GraphPad Inc., CA, USA).

### Recycling and degradation of the internalized WT and mutant FSHR

Postendocytic processing of the FSHR was assessed by the pulse-chase procedure previously described (Ulloa-Aguirre et al., 2013). In this assay, relative amounts of total recycled ^125^I-FSH [trichloroacetic acid (TCA)-insoluble radioactivity in the medium plus surface bound radioactivity] and degraded ^125^I-FSH (TCA-soluble radioactivity in the medium) are determined at frequent incubation times during 4 h, after allowing internalization of the receptor-ligand complex for 120 min. At each time point, recycled ^125^I-FSH is an indirect measurement of the amount of internalized receptor that was recycled back to the plasma membrane, whereas the amount of degraded ^125^I-FSH represents the fraction of the internalized receptor that was targeted to lysosomes and/or proteasomes for degradation. In some experiments, either 50 μM of MG132 (proteasome inhibitor), 100 nM of concanamycin A (lysosomal inhibitor) (Sigma) or both were added to the incubation medium after the initial 2 h incubation period.

### FSH-Stimulated ERK1/2 phosphorylation

Transfected cells were replated in 12-well-culture plates and tested for FSH-stimulated ERK1/2 phosphorylation (Tranchant et al., 2011). Briefly, after a 4 h preincubation period, 100 ng/ml recombinant FSH were added to cells and incubated for 0–120 min. At the end of the incubation period, cells were lysed in 2x Laemmly buffer and analyzed by Western blot. The membranes were incubated overnight at 4°C with anti-phospho-ERK1/2 (1:3000; Cell Signaling Technology Inc., Beverly, MA, USA) and then with secondary anti-mouse IgG horseradish peroxidase conjugate (Biosource). Signal was developed as described above. Equal protein loading was confirmed in a membrane reprobed with primary polyclonal antibody against total ERK2 (1:10,000; Santa Cruz Biotechnology Inc., Santa Cruz, CA, USA).

### Statistical analysis

Differences between means from ≥2 groups were analyzed employing either the unpaired Student's *t*-test or one-way analysis of variance (ANOVA) followed by *post-hoc t*-tests with Bonferroni correction. To analyze differences between the responses yielded by the internalization and recycling experiments, the areas under the curves (AUC) were calculated using GraphPad PRISM 4.0 (GraphPad Software, Inc., La Jolla, CA, USA) and the corresponding means compared as described above. Values and graphs shown are the means ± SD from three to five independent experiments. Probabilities of *p* < 0.05 were considered statistically significant.

## Results

### ^125^I-FSH binding, western blot analysis, and FSH-stimulated cAMP production

**Figure 1:**
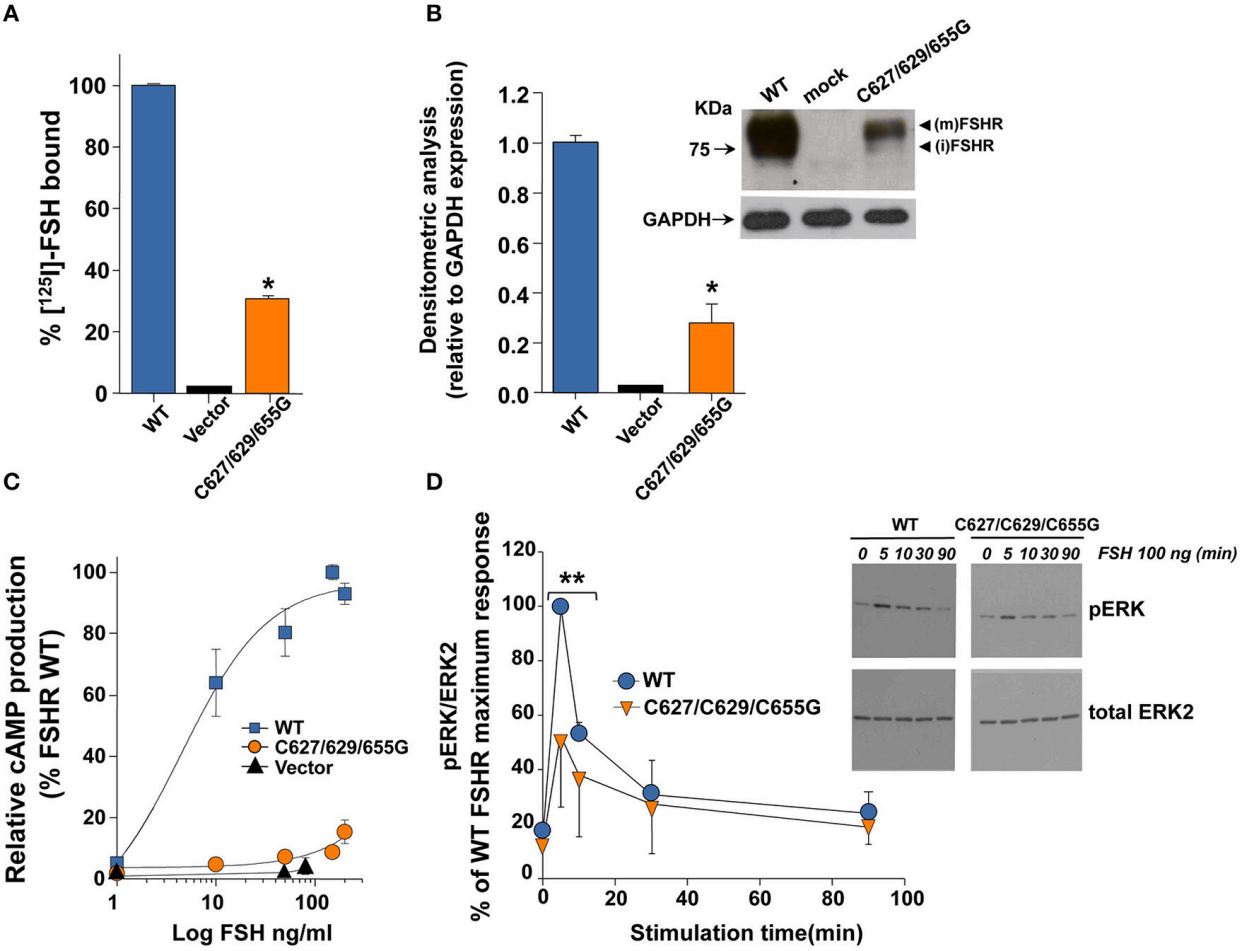
**Plasma membrane expression (A,B) and FSH-stimulated cAMP production and ERK1/2 phosphorylation (C,D) of the WT and triple mutant. FSHR**. **(A)** Specific ^125^I-labeled FSH binding to HEK-293 cells transiently expressing the WT or triple mutant FSHR. ^*^p < 0.01. **(B)** Plasma membrane expression of the triple mutant FSHR as disclosed by Western Blot analysis (relative to GAPDH expression, setting the WT FSHR at 1) (^*^p < 0.01). Inset, Relevant portion of an authoradiogram from an immunoblot of the WT FSHR and the triple mutant FSHR. GAPDH, Glyceraldehyde 3-phosphate dehydrogenase; (m): mature form of FSHR, (i) immature form of FSHR. **(C)** Relative cAMP production of HEK-293 cells transiently expressing the WT FSHR or the triple mutant FSHR, exposed to increasing doses of recombinant FSH during 18 h. ^*^p < 0.01. **(D)** Kinetics of ERK phosphorylation (pERK) induced by exposure of HEK-293 transiently expressing the WT or triple mutant FSHR to a fix dose (100 ng/ml) of recombinant FSH. In the densitometric analysis shown, the maximal WT FSHR-induced ERK phosphorylation was arbitrarily chosen as 100%. Inset: Representative immunoblot for FSH-induced ERK phosphorylation. ^**^p < 0.05.

We first analyzed the PM expression and intracellular signaling of the triple C627/629/655G FSHR mutant in order to test the functional impact of replacing the Cys residues that are palmitoylated in the WT FSHR with Gly. Compared to cells transfected with the WT FSHR cDNA plasmid, ^125^I-FSH binding to HEK-293 cells expressing the mutant receptor was significantly reduced at levels ~30% from those observed for the WT FSHR (Figure 1A). Western blot analysis of protein extracts from cells transfected with the mutant FSHR cDNA confirmed the limited PM expression levels of the mutant receptor as revealed by a ~70% reduction in intensity of the ~80 KDa band that represent the mature, PM-expressed, fully glycosylated FSHR (Ulloa-Aguirre et al., 2013; Figure 1B). Intracellular signaling mediated by the mutant FSHR also was attenuated as revealed by a markedly reduced cAMP production (Figure 1C) and, to a lesser extent, ERK1/2 phosporylation during the first 10 min of exposure to the agonist (Figure 1D).

In addition to the functional studies described above, we also applied computational modeling and MD simulation techniques to investigate the impact of the Cys to Gly substitutions on the conformational stability of the FSHR. For comparative purposes we additionally generated 20 ns long trajectories for a WT FSHR bearing non-palmitoylated Cys residues as well as for a mutant receptor with all Ctail cysteine residues replaced with alanine. The results of this *in silico* analysis are shown in Figures S1–S3. As shown in these figures, the conformation of the 7-transmembrane helices and the extra- and intracellular loops of the three FSHRs modeled remained relatively stable across the 20 ns trajectory simulated, as disclosed by the evolution of the corresponding root mean square deviation (RMSD) during the simulation time (Figure S1). In all FSHRs, the amino acid residues in positions 627 and 629 remained associated to the lipid bilayer modeled. The analysis of the local conformational changes along the protein chains during the last 4 ns of the simulation revealed similar local fluctations for the triple FSHR mutants (Cys to Gly and Cys to Ala mutants), including the amino-terminal end of the Ctail, where residues 627 and 629 are located, with larger fluctuations in the carboxyl-terminal end of all receptors simulated (Figure S2, upper panel). Interesting, the conformational fluctuations in the Ctail of the depalmitoylated WT FSHR simulated were larger than those exhibited by the FSHR mutants, emphasizing on the importance of S-acylation at Cys residues on the conformational stability of this particular domain (Figure S2, lower panels). Monitoring of the secondary structure elements (α-helices and β-strands) throughout the simulation trajectory confirmed the larger fluctuations of the carboxyl-terminal end of all simulated receptors and the relative stability of the α-helices, including the amino-terminal end of the Ctail (Figure S3).

In ensemble, these data indicate that substitution of the Cys residues at the FSHR Ctail with glycine impaired FSHR receptor expression and intracellular signaling as previously observed for the alanine-mutated receptor (Uribe et al., 2008), albeit with slight differences in the level of PM expression and agonist-stimulated intracellular signaling despite the similarities in magnitude of both conformational changes and regional fluctuations between the two receptor mutants.

### Digestion with endoglycosidase H of the WT and triple mutant FSHR

**Figure 2:**
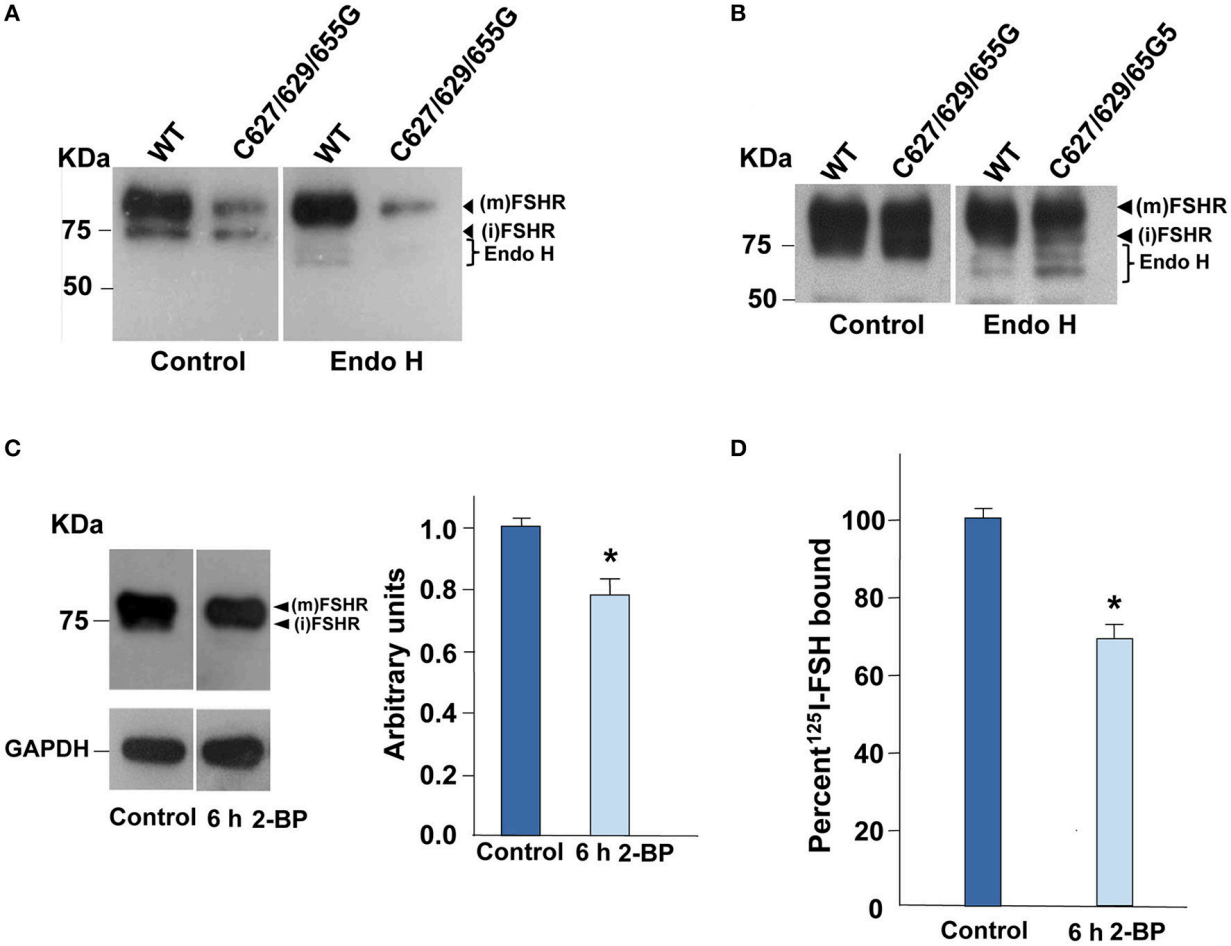
**Digestion of the FSHR with Endo H and plasma membrane expression upon exposure of HEK-293 cells expressing the WT FSHR to the palmitoylation inhibitor 2-bromopalmytate**. **(A)** Representative Western blot analysis of the WT and triple mutant FSHR before (control) and after treatment of protein extracts with Endo-H. A reduction in the intensity of the 75 KDa band but not in the 80 KDa band was observed as a result of Endo-H digestion; in **(B)** gels were uploaded with twice the amount of protein extracts from cells expressing the triple mutant FSHR, and anti-FSHR antibody-probed membranes were then overexposed to the radiographic film. **(C)** Representative immunoblot and densitometric analysis of the WT FSHR from cells treated with 50 μM 2-bromopalmitate or vehicle alone (DMSO, control). A reduction in the intensity of the band corresponding to the FSHR was found as disclosed by densitometric analysis (right panel). (m): mature form of FSHR, (i) immature form of FSHR. **(D)** The plasma membrane expression of the WT FSHR was also reduced as a result of the brief (6 h) exposure to the palmitoylation inhibitor, as revealed by receptor binding studies.^*^p < 0.05 vs. control.

To corroborate that, in fact, the 80 KDa band observed in the immunoblotting analysis of the C627/629/655G FSHR mutant described above corresponded to the fraction of mature FSHR that reached the PM, we performed Endo-H digestion of both the triple mutant and the WT FSHR. As shown in Figures 2A,B, incubation of protein extracts from cells transiently expressing the WT and mutant FSHR with Endo-H, resulted in a gel shift of the ~75 KDa band representing immature, incompletely processed FSHR down to bands with lower molecular weights, which correspond to more immature forms (Ulloa-Aguirre et al., 2013). In the case of the FSHRC627/629/655G, this shift was more clearly evident when gels were loaded with twice the amount of protein and overexposed (Figure 2B). These results indicated that replacement of cysteine residues at the Ctail of the FSHR did not influence on receptor glycosylation as the intensity of the PM-expressed, mature FSHR 80 KDa form in both the WT and triple mutant receptor remained unchanged after Endo-H exposure.

### WT FSHR PM expression in the presence of 2-bromopalmitate

Since in other GPCRs S-palmitoylation plays in important role in intracellular trafficking of the receptor from the endoplasmic reticulum-Golgi compartment to the PM (Qanbar and Bouvier, 2003), we assessed PM expression of the WT FSHR after incubation with the S-palmitoylation inhibitor 2-bromopalmitate (Davda et al., 2013) for 6 h. Exposure of cells expressing the WT FSHR to the irreversible palmitoylation inhibitor for 6 h, resulted in decreased PM expression of the receptor as disclosed by immunoblotting (~20% reduction in intensity of the 80 KDa band compared with the corresponding band from control cells; Figure 2C) and receptor binding (~30% decrease in specific ^125^I-FSH binding; Figure 2D). These data indicated that reduction or abolition of palmitoylation by either incubating in the presence of 2-bromopalmitate or mutating the cysteine residues present in the Ctail decreased PM expression of the FSHR, which secondarily could lead to reduced signaling response to agonist exposure.

### Internalization and β-arrestin 1 and 2 recruitment

**Figure 3:**
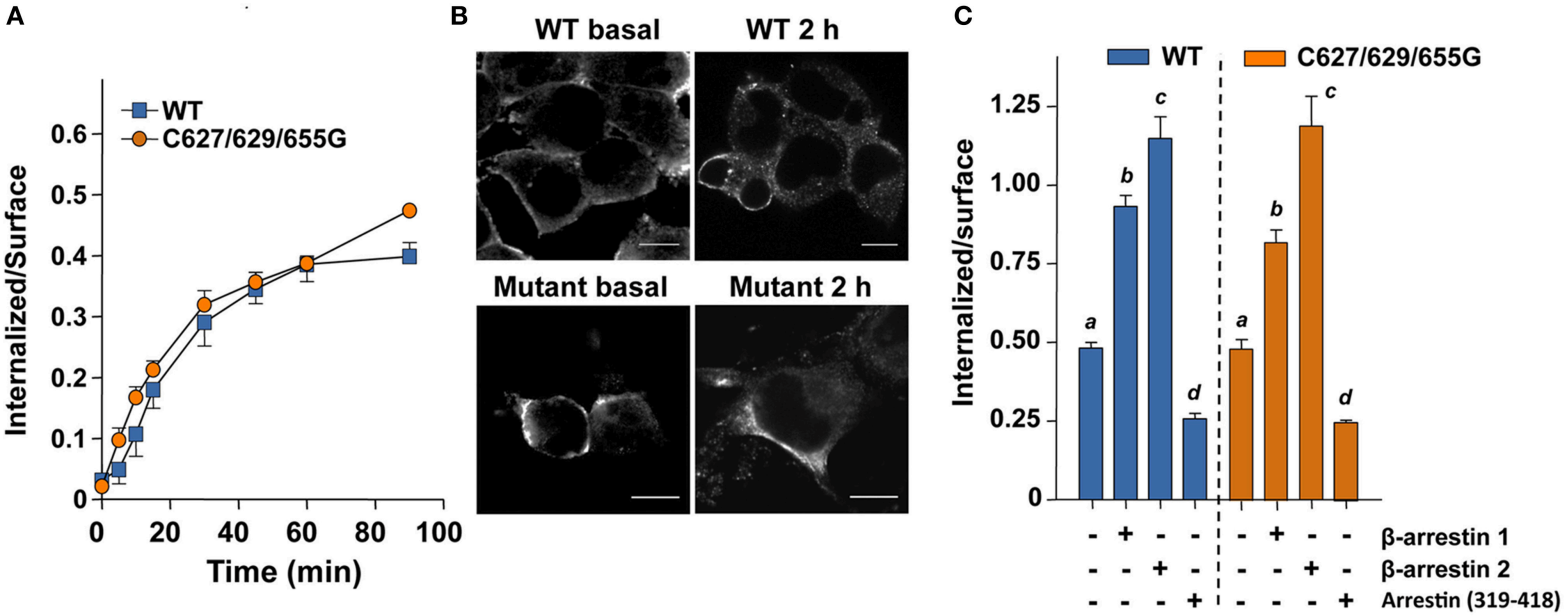
**FSH-stimulated internalization of ^125^I-FSH measured under non-equilibrium binding conditions in the absence (A,B) or presence of overexpressed β-arrestins (C)**. **(A)** Internalized (cell associated)/surface ^125^I-FSH ratio in cells transiently expressing either the WT or the triple mutant FSHR as a function of time. The internalization kinetics were similar between the two FSHRs. **(B)** Internalization of WT and mutant FSHR before (basal) and after 2-h exposure to recombinant FSH as assessed by confocal microscopy. The intracellular fluorescence increased in both HEK-293 cells expressing the WT and the triple mutant FSHR after exposure to agonist for 2 h. This was more evident in the case of the WT FSHR due to its much higher expression compared to that exhibited by the triple mutant receptor (See Figures 1A,B). **(C)** Agonist-stimulated internalization of the ^125^I-FSH/FSHR complex in cells transiently co-transfected with either the WT or the mutant FSHR and β-arrestin 1, β-arrestin 2 or dominant-negative arresting (319–418). Under these conditions the internalization of both receptors were virtually the same. Different letters among bars indicate statistical differences (<0.05) within each FSHR. See supplementary information (Figure S4) for details on the fluorescence analysis of the images shown in **(B)**.

Next we examined the effects of replacing the cysteine residues of the FSHR Ctail on agonist-stimulated downward trafficking of the PM-expressed receptor. Given that agonist-induced desensitization is followed by internalization of the agonist-bound receptor and that in both processes β-arrestins play an important role, we first studied the internalization kinetics of the mutant FSHR and its regulation by β-arrestin 1 and 2. As illustrated in Figure 3A, the internalization kinetics of the triple FSHR mutant [expressed as the relationship between the amount (cpms) of internalized and surface-bound ^125^I-FSH at time 90 min] was comparable to that shown by the unmodified receptor. Confocal microscopy of cells transiently expressing the FSHR before and 2 h after FSH exposure confirmed that the mutant FSHR internalized with similar efficiency to its WT counterpart (Figure 3B and Figure S4). In basal conditions, fluorescence could be predominantly identified at the cell periphery, whereas 2 h after agonist exposure there was a strong increase in fluorescence inside the cells (Figure S4B, lower panel). Figure 3C shows the internalization of ^125^I-FSH in cells expressing the WT and mutant FSHR after 90 min exposure to FSH in cells overexpressing β-arrestin-1 and -2 or β-arrestin 319–418. Over-expression of either β-arrestin 1 or 2, led to a significant increase in the internalization rate of both the WT and mutant FSHRs, whereas overexpression of the dominant-negative form of β-arrestins reduced internalization of both receptors by nearly 50%. The magnitude of changes in FSHR internalization under these conditions was similar for the WT and the mutant FSHR. These results indicated that the replacement of all Ctail cysteine residues with glycine, preventing palmitoylation of the FSHR, neither significantly altered endogenous β-arrestin recruitment nor agonist-stimulated internalization. Further, although the magnitude of FSH-stimulated ERK phosphorylation in cells transfected with the triple mutant FSHR was modest (due to low PM expression), the pattern of FSH-stimulated ERK1/2 activation, which depends on both Gα_*s*_/PKA- and β-arrestins 1 and 2-dependent pathways (Kara et al., 2006; Shenoy et al., 2006), was virtually indistinguishable between cells expressing the WT or mutant FSHR (Figure 1D). Mock-transfected HEK-293 cells did not show any FSH-induced pERK (not shown).

### Degradation and recycling of the FSH/FSHR

**Figure 4:**
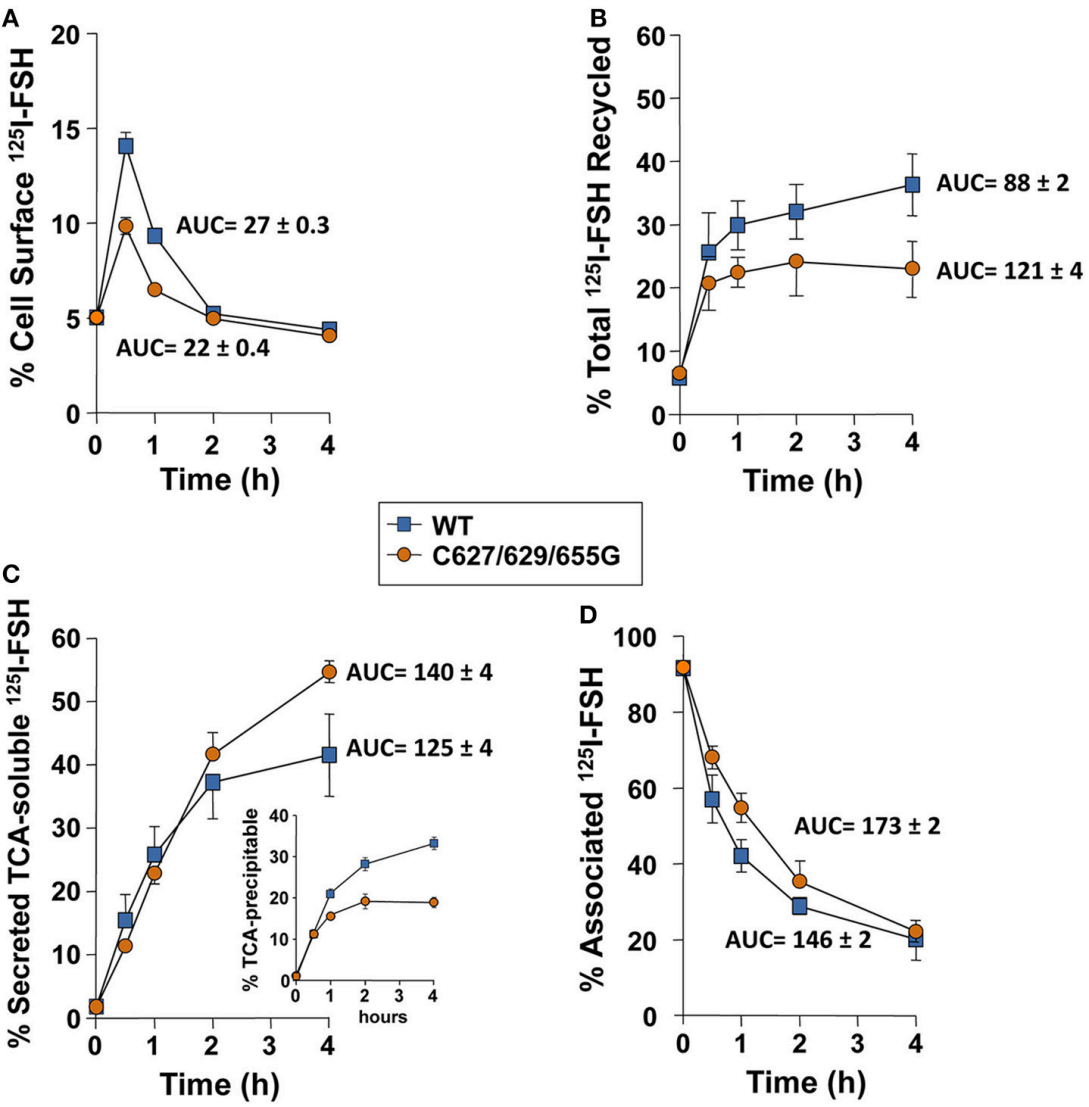
**Kinetics of recycling back to the plasma membrane of the WT and triple mutant FSHR after agonist-provoked internalization**. **(A)** Cell surface ^125^I-FSH bound to the recycled FSHR throughout the second, 4 h incubation (see Materials and Methods). The relative amount of cell surface ^125^I-FSH/mutant FSHR complex was less compared to that shown by the WT receptor (see Results Section). **(B)** Total ^125^I-FSH/FSHR complex recycled back to the plasma membrane (cell surface ^125^I-FSH plus TCA precipitable ^125^I-FSH), following exposure of HEK-293 cells transiently expressing the WT or mutant FSHR to agonist. Total recycled FSH/mutant FSHR complex was decreased compared to the FSH/WT FSHR complex (see Results Section). **(C)** TCA-soluble (degraded) radioactivity recovered from the culture medium throughout the second, 4 h incubation. Less soluble radioactivity was recovered at time 4 h in media from cells transfected with the mutant receptor; meanwhile, more TCA precipitable (undegraded) ^125^I-FSH was recovered in the media from cells expressing the WT FSHR (inset). **(D)** Cell-associated ^125^I-FSH recovered during the second incubation.

The fate of FSHRC627/629/655G following agonist-provoked internalization was studied employing the pulse-chase procedure described in Material and Methods. Figure 4 shows that cells transiently expressing the cysteine-replaced Ctail FSHR recycled internalized ^125^I-FSH back to the plasma membrane less efficiently than those expressing the WT receptor (Figures 4A,B; AUC of total ^125^I-FSH recycled/240 min in cells transfected with FSHRC627/629/655G: 88 ± 2 vs. 121 ± 4 in cells expressing the WT receptor; *p* < 0.05). Accordingly, more secreted but degraded, TCA-soluble ^125^I-FSH (which reflects the fraction of the internalized receptor that was targeted to lysosomes and/or proteasomes) was recovered from the medium of cells transfected with the FSHRC627/629/655G (AUC of TCA-soluble ^125^I-FSH fraction/240 min: 140 ± 4) than from those expressing the WT receptor (^125^I-FSH/240 min: 125 ± 4; *p* < 0.05) (Figure 4C), whereas the opposite was observed for the TCA-precipitable ^125^I-FSH fraction (Figure 4C, inset). Finally, the amount of cell associated ^125^I-FSH/240 min was significantly higher in cells transfected with the triple Cys FSHR mutant (AUC/240 min: 173 ± 2) than in those transfected with the WT receptor (AUC/240 min: 146 ± 2; *p* < 0.05; Figure 4D). Nevertheless, at the end of the incubation period (4 h), the fraction of ^125^I-FSH that remained cell associated was the same for both WT- and FSHR C627/629/655G-transfected cells; this result correlated with the data depicted in Figures 4A–C showing higher amounts of WT receptor recycling back to the PM and, conversely, higher amounts of degraded ^125^I-FSH and secreted as TCA-soluble ^125^I by cells transfected with the triple mutant receptor.

**Figure 5:**
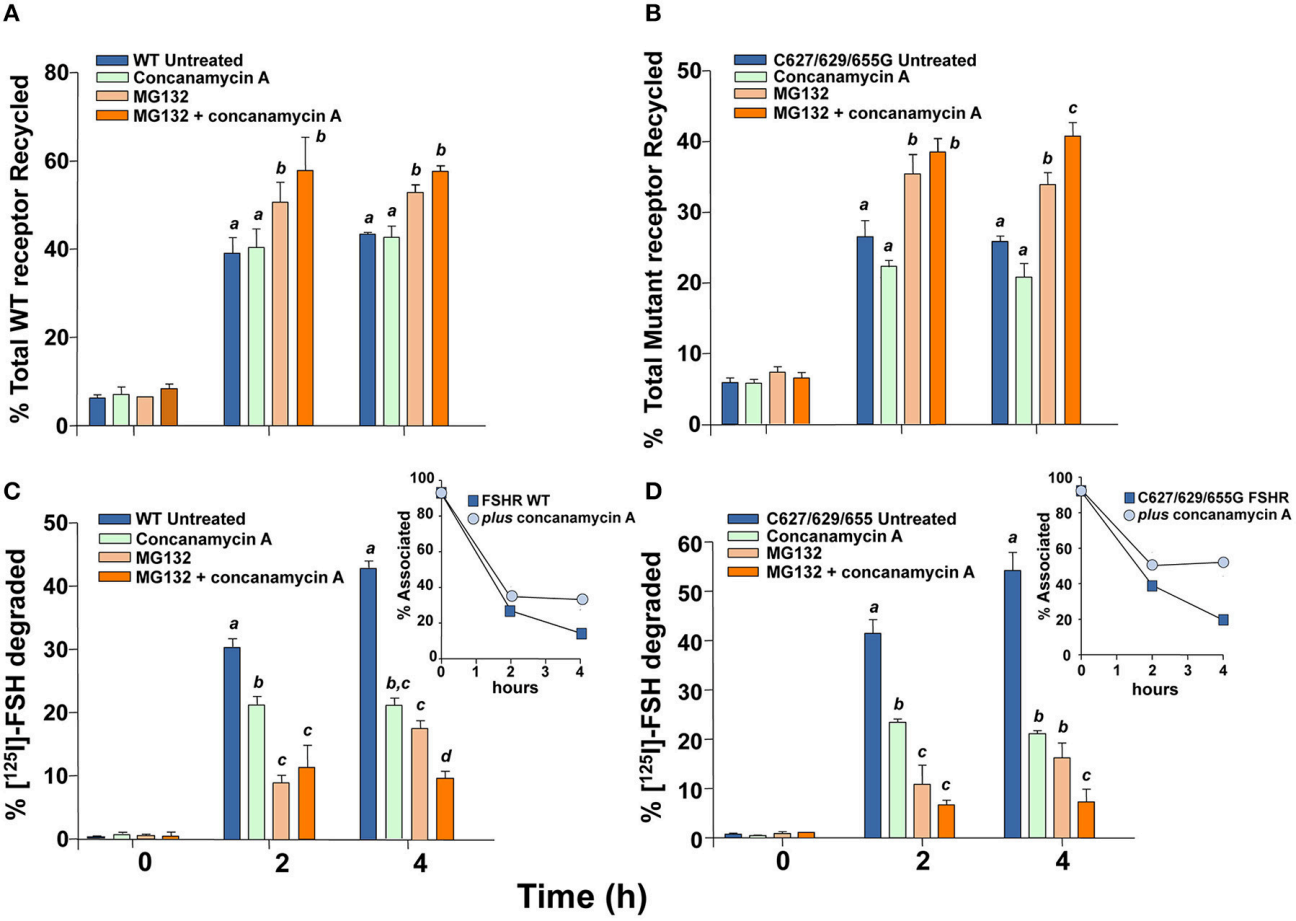
**Recycling of the WT and triple mutant FSHR after agonist-provoked internalization (time zero), in the presence and absence of concanamycin A (lysosomal inhibitor), MG132 (proteasome inhibitor) or both**. Total recycled FSH/FSHR complex increased upon exposure to MG132 but not concanamycin A **(A,B)**. Meanwhile, the fraction of TCA soluble (degraded) ^125^I-FSH in media from cells expressing the WT **(C)** or mutant FSHR **(D)** decreased in the presence of either MG132, concanamycin A or both (see Results). Different letters among bars indicate statistical differences (<0.05) within each time (0, 2, or 4 h).

**Figure 6:**
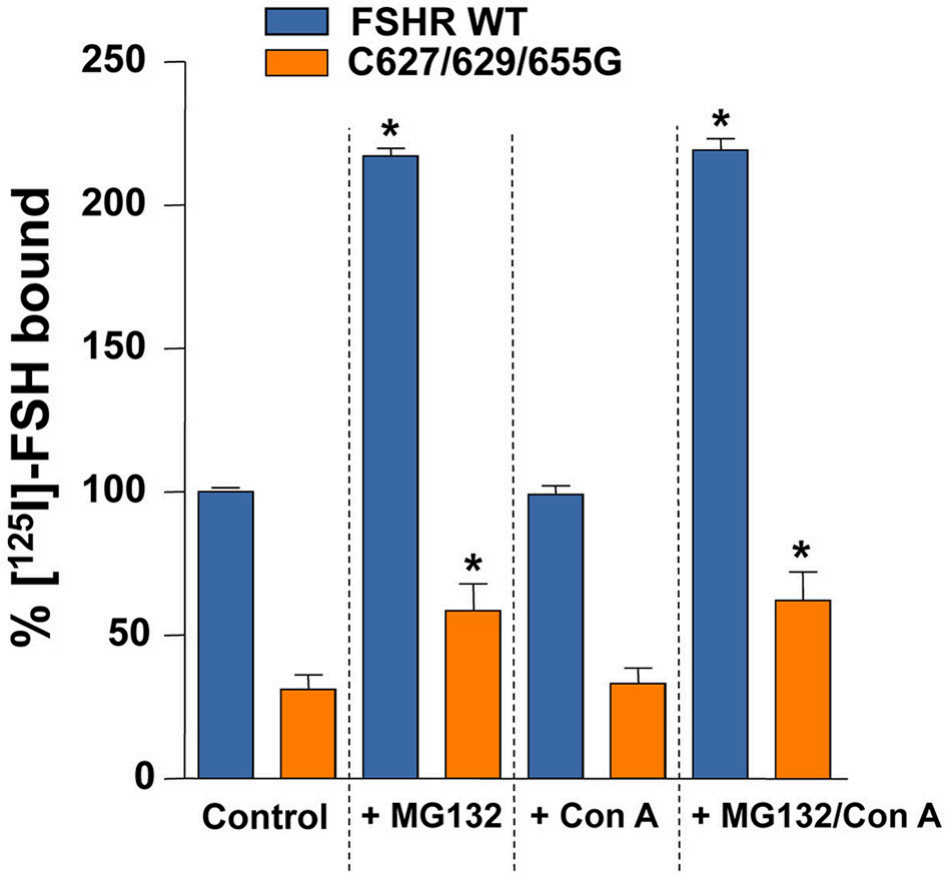
**Specific ^125^I-FSH binding in HEK-293 cells expressing the WT or the triple mutant FSHR at the end of the second, 4 h incubation period of the recycling experiments, in the absence (control) or presence of MG132, concanamycin A (Con A), or both**. ^*^p < 0.05 vs. control for the same FSHR.

To confirm that FSHRC627/629/655G recycled less to the PM than the WT receptor after agonist-stimulated internalization as a consequence of increased proteasomal/lysosomal degradation, further recycling experiments were performed in the absence or presence of inhibitors of lysosome and/or proteasome degradation. As shown in Figures 5 and 6, addition of the lysosomal inhibitor concanamycin A failed to increase the amount of recycled FSHR in cells expressing either the WT or the mutant FSHR (Figures 5A,B). It should be noticed that although exposure to the lysosome inhibitor provoked a marked decrease in the amount of degraded ^125^I-FSH, the fraction of cell associated hormone was higher than in unexposed conditions, particularly at 4 h (Figures 5C,D). By contrast, exposure to the proteasome inhibitor MG132 for 2 and 4 h, resulted in a significant increase in the fraction of recycled WT and mutant FSHR and a parallel decrease in the secreted ^125^I-FSH TCA soluble fraction. Further, the increase in recycled FSHR (WT and mutant) in response to proteasome inhibitor correlated with a significant increment in specific ^125^I-FSH binding after 4 h of incubation (Figure 6). For both FSHRs, specific ^125^I-FSH binding in cells exposed to MG132 was twice as much as that exhibited by cells unexposed to the inhibitor, whereas in cells exposed to concanamycin A alone there were no changes in specific agonist binding. Nevertheless, the more than two-fold increase in agonist binding after exposure to MG132, might reflect not only inhibition of proteasomal degradation after internalization but also during the upward trafficking of the receptor from the endoplasmic reticulum/Golgi compartments to the PM. These results indicate that abrogation of FSHR palmitoylation by replacing all Ctail cysteine residues, does not affect internalization of the FSH/FSHR complex, but favors sorting of the complex to the degradation pathway.

## Discussion

Given their particular physicochemical properties, cysteine residues in the carboxyl-terminus of GPCRs are susceptible to several post-translational modifications, including formation of disulfide bonds and lipid modifications (Zhou et al., 2014). Disulfide bonds in the cytosolic compartment are quite unstable due to the reducing environment, whereas S-acylation with palmitic acid is a well-conserved and prevalent posttranslational modification among members of the GPCR superfamily (Qanbar and Bouvier, 2003). Besides facilitating association of the Ctail with the plasma membrane, promoting formation of a fourth cytoplasmic loop, several studies have documented other functional roles for palmitoylation in GPCRs, which will vary depending on the particular receptor, and that include modulation of receptor conformation, coupling to effectors, regulation of intracellular trafficking and PM localization, desensitization, and postendocytic processing (Bouvier et al., 1995; Qanbar and Bouvier, 2003; Resh, 2006; Linder and Deschenes, 2007). In the case of glycoprotein hormone receptors, mutation of Cys699 with alanine, a potential site of palmitoylation in the TSHR, has been shown to slow upward intracellular trafficking (Tanaka et al., 1998), whereas in the LHR replacement of two contiguous cysteine residues (621 and 622) with glycine did not affect PM expression of the receptor but led to a marked increase in ligand-stimulated internalization and decreased recycling back to the PM.

Herein, we mutated all cysteine residues present in the Ctail of the human FSHR. We have previously shown that all three cysteine residues present in this domain are palmitoylated (Uribe et al., 2008). In fact, alanine substitution of Ctail cysteine residues led to complete abrogation of palmitoylation and decreased specific ^125^I-FSH binding, cell surface PM expression, and agonist-stimulated intracellular signaling. Here we found that in cells expressing the FSHRC627/629/655G, PM expression of the mature form of the receptor was drastically reduced, thus confirming our previous findings (Uribe et al., 2008). Further, we observed that a brief (6 h) exposure to 2-bromopalmitate, was accompanied by a ~20% decrease in the amount of the mature form of FSHR protein and concomitantly in ~30% ^125^I-FSH binding. These data suggest that abrogation of palmitoylation of the FSHR by removing all Ctail cysteine residues, limits the traffic of the receptor to the PM, as documented for other GPCRs (Gao et al., 1999; Petaja-Repo et al., 2006; Adams et al., 2011). Nevertheless, our data do not allow to unambiguously identify whether the limited cell surface PM expression of the mutant FSHR was primarily due to the lack of palmitoylation *per se* or to changes in the conformation of the Ctail domain as replaced residues in positions 627 and 629 are near the F(X)_6_LL sequence, which is closely involved in the transport of several GPCRs (including the FSHR) to the PM (Duvernay et al., 2004; Timossi et al., 2004). Further, MD simulation of mutant FSHRs with individual alanine replacements in Ctail cysteine residues revealed that mutants involving position 629 yielded the most unstable structures, with the triple mutant structure resulting in a highly unstable conformer (Uribe et al., 2008), more susceptible to rapid degradation. In this vein, it was interesting to find that the conformational changes exhibited by the Ctail of the FSHR Gly^3^ mutant were similar to those observed in the Ala^3^ FSHR and that exposure to the proteasome inhibitor MG132 led to a more than two-fold increase in FSH binding to both WT and mutant receptor, which may be an indication of the impact of proteasomal degradation on both upward and downward trafficking of the FSHR. In fact, it has been shown that palmitoylation-deficient mutants of some GPCRs, such as the human A1 adenosine receptor, are more prone to rapid proteolytic degradation (Gao et al., 1999). According to this information, we may conclude that the significant reduction in PM expression of Ctail Cys mutant FSHRs (Uribe et al., 2008 and present study) in basal conditions may be due to several factors, including abrogation of palmitoylation, receptor misfolding, and increased proteasomal degradation, all of which may limit trafficking of the receptor from the endoplasmic reticulum and/or Golgi compartments to the plasma membrane.

We found that FSH-stimulated phosphorylation of ERK1/2 followed a similar kinetics when mediated by either the WT or FSHRC627/629/655G, that is, a rapid and transient increase in pERK peaking 5 min after agonist exposure, which mainly depends on Gαs activation, followed by an increased level above basal during the ensuing 30–60 min, which is G protein-independent but β-arrestin-dependent (Kara et al., 2006). The finding of a stronger effect of the mutations on FSH-stimulated cAMP production than in ERK1/2 phosphorylation, might reflect the contribution of endogenous β-arrestins to activation of this kinase. In fact, we found that the endogenous β-arrestin recruitment by the triple mutant FSHR was virtually identical to that shown by the WT receptor. These findings suggest that mutation of all cysteine residues present in the Ctail of the FSHR, although adversely affected the cAMP response to agonist, did not parallely alter with the same intensity the efficacy to elicit ERK phosphorylation via the β-arrestin-dependent pathway, despite its low PM expression levels. A similar dissociation in response to agonist has also been reported for the misfolded A189V FSHR mutant, which associates with intracellular retention of the FSHR and severely impaired cAMP production (Tranchant et al., 2011). Whether this difference in response to agonist is due to biased signaling of the mutant FSHR or to conditional bias secondary to alterations in the stoichiometry of receptor-interacting partners (Landomiel et al., 2014) remains to be further investigated. In this regard, studies in other GPCRs, such as the endothelin A receptor documented that depalmitoylation was associated to biased signaling, with a selective loss in its ability to couple to Ca^2+^ mobilization without affecting the Gs/cAMP pathway (Horstmeyer et al., 1996).

In the present study, we provided new information on the role of Ctail cysteine residues in downward trafficking of the FSHR, particularly on the kinetics of its postendocytic processing after agonist-stimulated internalization. Most investigators agree in that palmitoylation influence the downward trafficking of transmembrane proteins, including internalization, endocytosis, recycling, and degradation (Chini and Parenti, 2009). In the case of the LHR, it has been shown that prevention of palmitoylation by mutating the Ctail Cys residues to glycine, leads to both increase in agonist-stimulated internalization and reduction in the efficiency of recycling of the internalized receptor back to the PM (Munshi et al., 2005). Since palmitoylation-deficient GPCRs, including the LHR, may be hyperphosphorylated (Munshi et al., 2001), it has been speculated that decreased recycling of the depalmitoylated LHR could be due to resistance to endosomal dephosphorylation of the hyperphosphorylated receptor (Munshi et al., 2005). Here, we found that recycling of the internalized triple mutant FSHR back to the PM was considerably reduced. The observation that the internalization rate and its dependency to β-arrestins was comparable for the FSHRC627/629/655G and WT receptors, suggesting that the mutant FSHR was not hyperphosphorylated, makes resistance to dephosphorylation a less plausible explanation for the increased degradation of the FSHR mutant. Rather, it appears that the decreased recycling of FSHRC627/629/655G was due to failure of the altered Ctail to correctly interact with the recycling endosome machinery or to properly interact with proteins associated with recycling to the PM [e.g., the GPCR interacting protein Na^+^-H^+^ exchange regulatory factor or NHERF1, which promotes recycling of the β_2_-adrenergic receptor and the κ-opioid receptor (Cao et al., 1999; Li et al., 2002)], thereby favoring sorting of the mutant receptor to the lysosomal/proteasomal degradation pathway. Since palmitoylation of Cys655 appears to be involved in the internalization of the FSHR (Uribe et al., 2008), it is also possible that absence of palmitoylation in this location may promote decreased recycling to the PM given that this particular cysteine residue is located near a downstream, short amino acid sequence determinant for postendocytic processing of the FSHR (Krishnamurthy et al., 2003b). Whatever the mechanism(s) by which the absence of S-palmitoylation increase degradation of the ligand/receptor complex, the present findings unveil additional structural components (i.e., S-palmitoyable cysteine residues) involved in efficient recycling of the internalized FSHR back to the PM.

Similarly to findings in the β_2_-adrenergic receptor and the μ- and δ-opioid receptor (Chaturvedi et al., 2001; Shenoy et al., 2001), our experiments showed that the increased degradation of the triple mutant FSHR was counteracted by proteasomal inhibition, thus suggesting that the ubiquitination-proteosomal degradation pathway is primarily involved in FSHR degradation after agonist-stimulated internalization. In fact, it has been shown that the WT FSHR is ubiquitinated and that proteasomal inhibitors increase cell surface residency of this receptor (Cohen et al., 2003), as it was observed in the present study. This finding contrasts with the results from incubations in the presence of concanamycin A, in which exposure to this lysosomal inhibitor led to increased accumulation of internalized hormone due to a decrease in hormone degradation but without modifying the total recycled receptor or hormone/receptor complex to the cell surface. This accumulation of internalized hormone might be due to perturbation in the flux through the proteasomes in the presence of the lysosomal disruptors, as it has been observed in other ubiquitinated proteins (Korolchuk et al., 2009; Qiao and Zhang, 2009). Due to the pulse-chase strategy employed to analyze the postendocytic processing of the internalized ^125^I-FSH/FSHR complex, it is not possible to discern whether the ^125^I-FSH remaining in the cell associated fraction (presumably in endosomal compartments) in the presence of concanamycin A corresponds to ^125^I-FSH complexed with or dissociated from the FSHR, which afterward recycled back to the PM. Nevertheless, the finding that specific ^125^I-FSH binding in the presence or absence of concanamycin A was virtually the same, indicates that the radioactivity remaining in the cell associated fractions rather corresponded to FSH complexed with the FSHR.

It was interesting to find that the combination of concanamycin A with MG132 further increased the recycled ^125^I-FSH fraction and concomitantly decreased that corresponding to the degraded fraction. This finding suggests that lysosomal degradation still may have a place in the postendocytic processing of the FSHR.

In summary, the results presented herein, demonstrate that replacement of cysteine residues with glycine in the Ctail of the FSHR markedly decreased both upward trafficking of the receptor to the PM as well as its recycling back to the PM following agonist-stimulated internalization, without modifying the internalization kinetics of the FSH/FSHR complex. These alterations in FSHR trafficking provoked by deprivation of cysteine residues at the Ctail, may be due to the absence of S-acylation or to the conformational alterations in this particular domain provoked by the mutations. These findings contrast with those reported for the structurally related LHR, in which replacement of Ctail cysteine residues (preventing palmitoylation of the receptor), did not significantly modify the upward trafficking of the receptor but markedly altered the internalization kinetics and postendocytic processing of the cysteine-lacking receptor (Munshi et al., 2001, 2005). Collateraly, the data also suggest that the ubiquitin/proteasome pathway operates upstream of trafficking to lysosomes to regulate the postendocytic processing of the human FSHR.

## Author contributions

BM-N, MP-S, JC-B, TZ, AA-R, and NG: performed the experiments, analyzed data, reviewed drafts, and approved final version of the manuscript. PC-G: Designed the experiments and performed the data analysis, reviewed, and analyzed data, approved final version. JM-M: performed the internalization analysis. EJ-V: performed the studies *in silico.* ER: Designed experiments, analyzed the data, reviewed drafts and approved final version of the manuscript. AU-A: Designed experiments, analyzed the data, interpreted data, and wrote the manuscript.

## Funding

This work was supported by the Consejo Nacional de Ciencia y Tecnología (CONACyT, Mexico) grants 86881, 240619 (to AU-A), and 78824 (to PC-G), by the Coordinación de la Investigación Científica, UNAM (to AU-A, JM-M, JC-B and TZ), and by grant FIS/IMSS/PROT/532 from the Instituto Mexicano del Seguro Social, Mexico (to AU-A).

### Conflict of interest statement

The authors declare that the research was conducted in the absence of any commercial or financial relationships that could be construed as a potential conflict of interest.
