# Supplementary material for: Role of Cysteine Residues in the Carboxyl-Terminus of the Follicle-Stimulating Hormone Receptor in Intracellular Traffic and Postendocytic Processing
Source: Front Cell Dev Biol. 2016 Jul 20;4:76. doi: 10.3389/fcell.2016.00076 (PMC4951517; doi:10.3389/fcell.2016.00076)
Supplement: Supplementary file 1 [file Image1.pdf]

Supplementary Figure S1

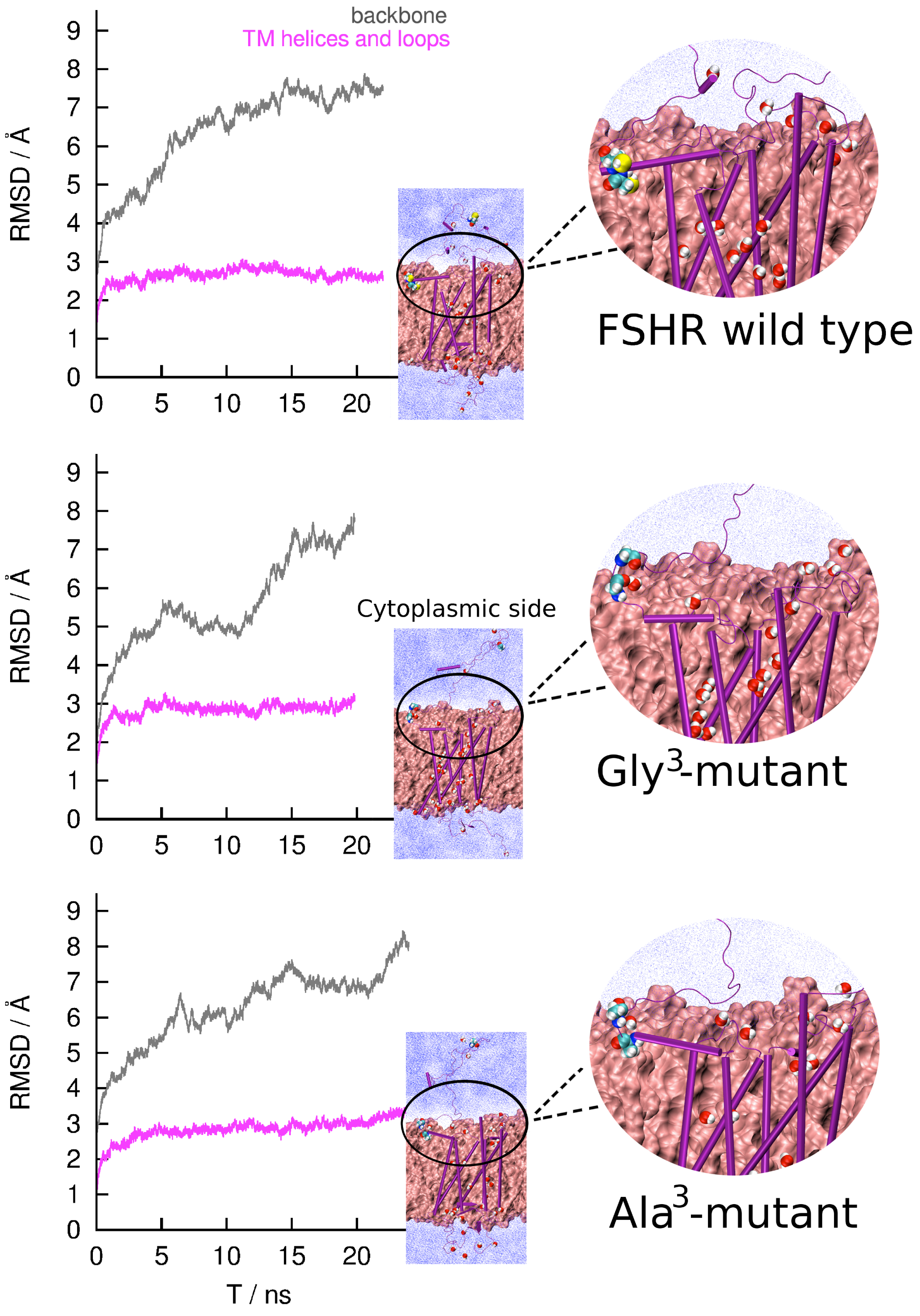

Figure S1. Root mean square deviation (RMSD) plots (in Å) for the WT FSHR model and the Gly[3] and Ala[3] FSHR mutants . The changes in RMSD showed by grey solid lines included the amino- and carboxyl-terminus of the modeled FSHRs; RMSDs reached almost ~8 Å (gray solid line) because of large conformational changes in the backbone of the receptors. In contrast, RMSD values of ~3 Å calculated for the transmembrane helices and loops (magenta solid line), indicate that the transmembrane structures were well preserved in the lipid bilayer environment during the simulation time. The insets display the snapshot of the last protein configuration in the lipid bilayer after 20 ns of MD simulation, with the lipids shown in pink surface, the protein as purple cylinders, the interhelical water molecules as spheres (oxygen in red and hydrogen in white), and solvent water as a blue background. The zoom at the cytoplasmic side of the receptor displays a similar context for the Cys, Gly and Ala627 and 629 residues, which are located at the interface between the bilayer and the aqueous region. The amino acid residue 655, located in the aqueous region, is away from the bilayer interface.
