# Supplementary material for: Role of Cysteine Residues in the Carboxyl-Terminus of the Follicle-Stimulating Hormone Receptor in Intracellular Traffic and Postendocytic Processing
Source: Front Cell Dev Biol. 2016 Jul 20;4:76. doi: 10.3389/fcell.2016.00076 (PMC4951517; doi:10.3389/fcell.2016.00076)
Supplement: Supplementary file 2 [file Image2.pdf]

Supplementary Figure S2

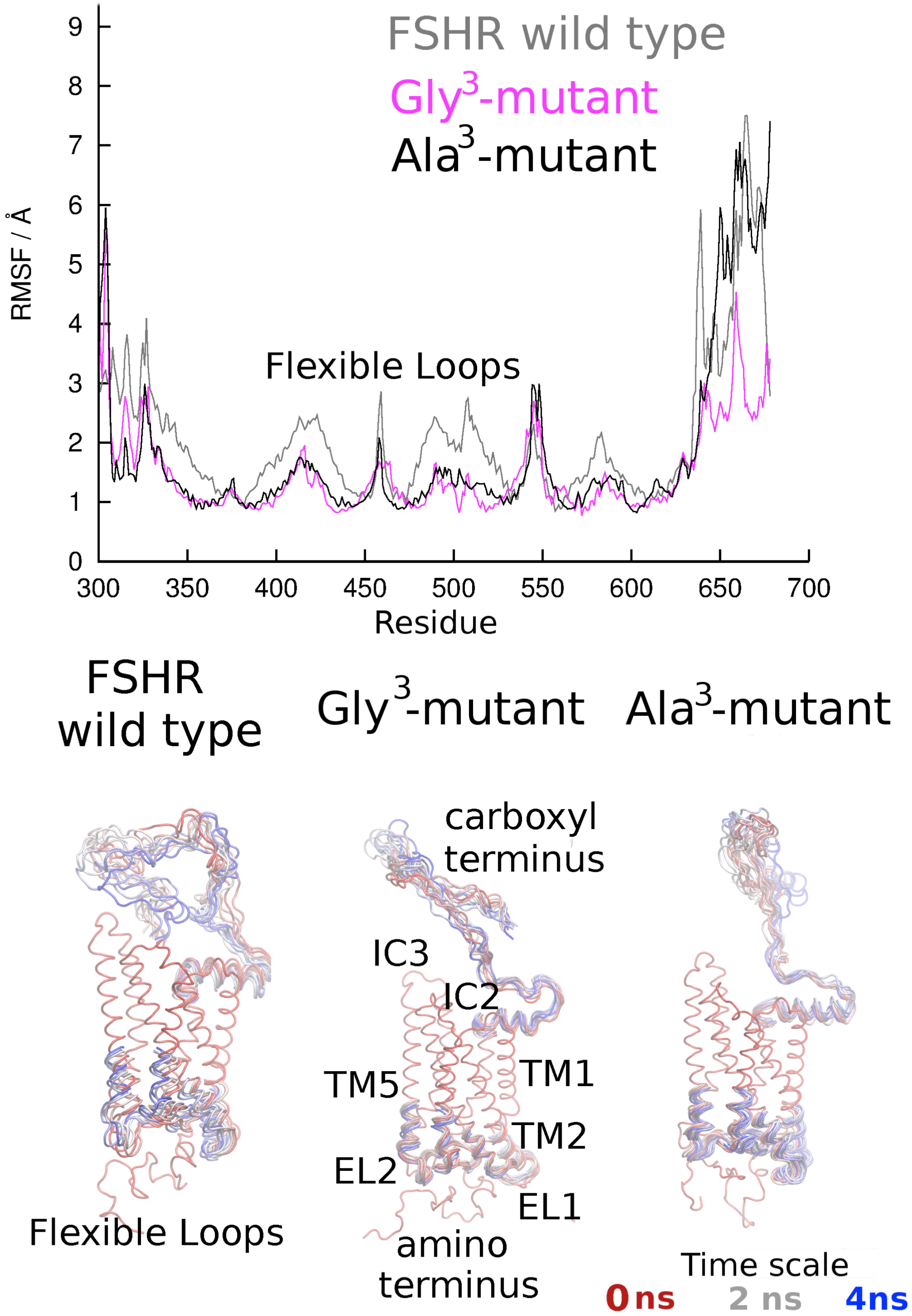

Figure S2. The upper plot shows the root mean square fluctuations (RMSF, in Å) for the WT FSHR, and the Gly^3 and Ala^3 mutants. The calculations were performed on the last 4 ns of the simulation in time frames stored every 50 ps. Higher values correspond to protein regions moving with larger amplitudes, such as the interhelical loops and the carboxyl- and amino-terminus. The dynamics along the protein sequence showed similar profiles. Overlays of the protein conformation at different simulation times are displayed in the bottom structures. The WT FSHR structure showed the largest conformational changes in the carboxyl-terminus and largest flexibility of the extracellular loops (EL). The higher flexibility of the extracellular loops of the WT FSHR explains the larger RMSF values calculated upon comparison against the Gly^3 and Ala^3 mutans.
