# Supplementary material for: Role of Cysteine Residues in the Carboxyl-Terminus of the Follicle-Stimulating Hormone Receptor in Intracellular Traffic and Postendocytic Processing
Source: Front Cell Dev Biol. 2016 Jul 20;4:76. doi: 10.3389/fcell.2016.00076 (PMC4951517; doi:10.3389/fcell.2016.00076)
Supplement: Supplementary file 3 [file Image3.pdf]

Supplementary Figure S3

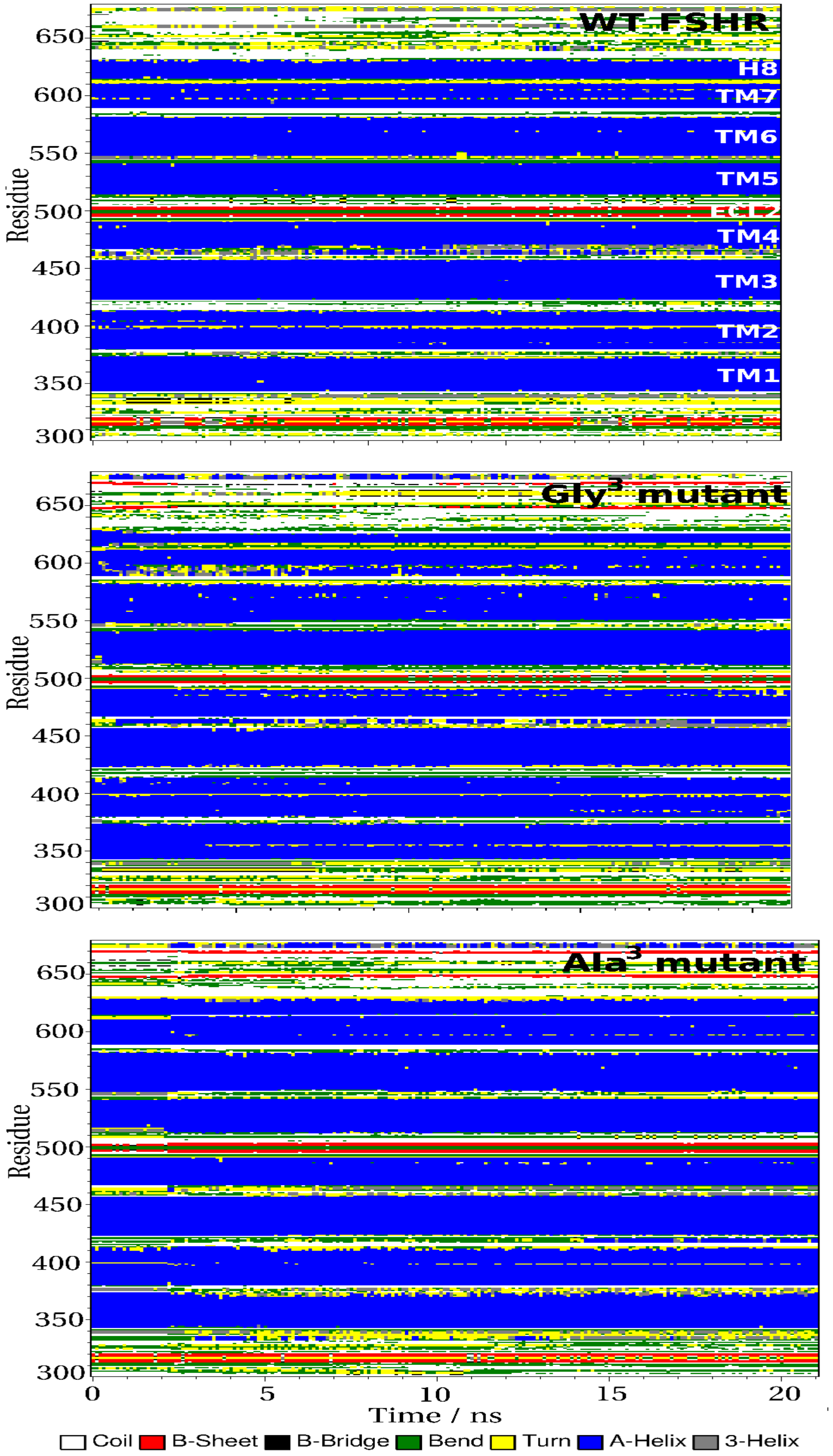

Figure S3. Secondary structure analysis of the WT FSHR and the Gly^3 and Ala^3 FSHR mutants. All protein structures exhibited stable seven transmembrane α-helices (TM1-TM7) across the lipid bilayer and a helix eight (H8) parallel to the bilayer at the intracellular interface. In all structures, stable β-sheet structures were observed in regions Asp317 to Asp320 and Met 495 to Ile505 in the amino-terminus and the second extracellular loop, respectively. The carboxyl-terminus was highly dynamic in all structures, with sporadic interconversion between α-helices and 3-helices and turns. At the secondary structure level, within ~20 ns of MD simulation, WT and triple mutants displayed similar profiles. A-Helix: α-helix (helix every 4 amino acid residues); 3-Helix (helix every 3 residues); B-Sheet: β-sheet; B-Bridge: β-bridge (single pair β-sheet hydrogen bond formation).
