# Supplementary material for: Role of Cysteine Residues in the Carboxyl-Terminus of the Follicle-Stimulating Hormone Receptor in Intracellular Traffic and Postendocytic Processing
Source: Front Cell Dev Biol. 2016 Jul 20;4:76. doi: 10.3389/fcell.2016.00076 (PMC4951517; doi:10.3389/fcell.2016.00076)
Supplement: Supplementary file 4 [file Image4.pdf]

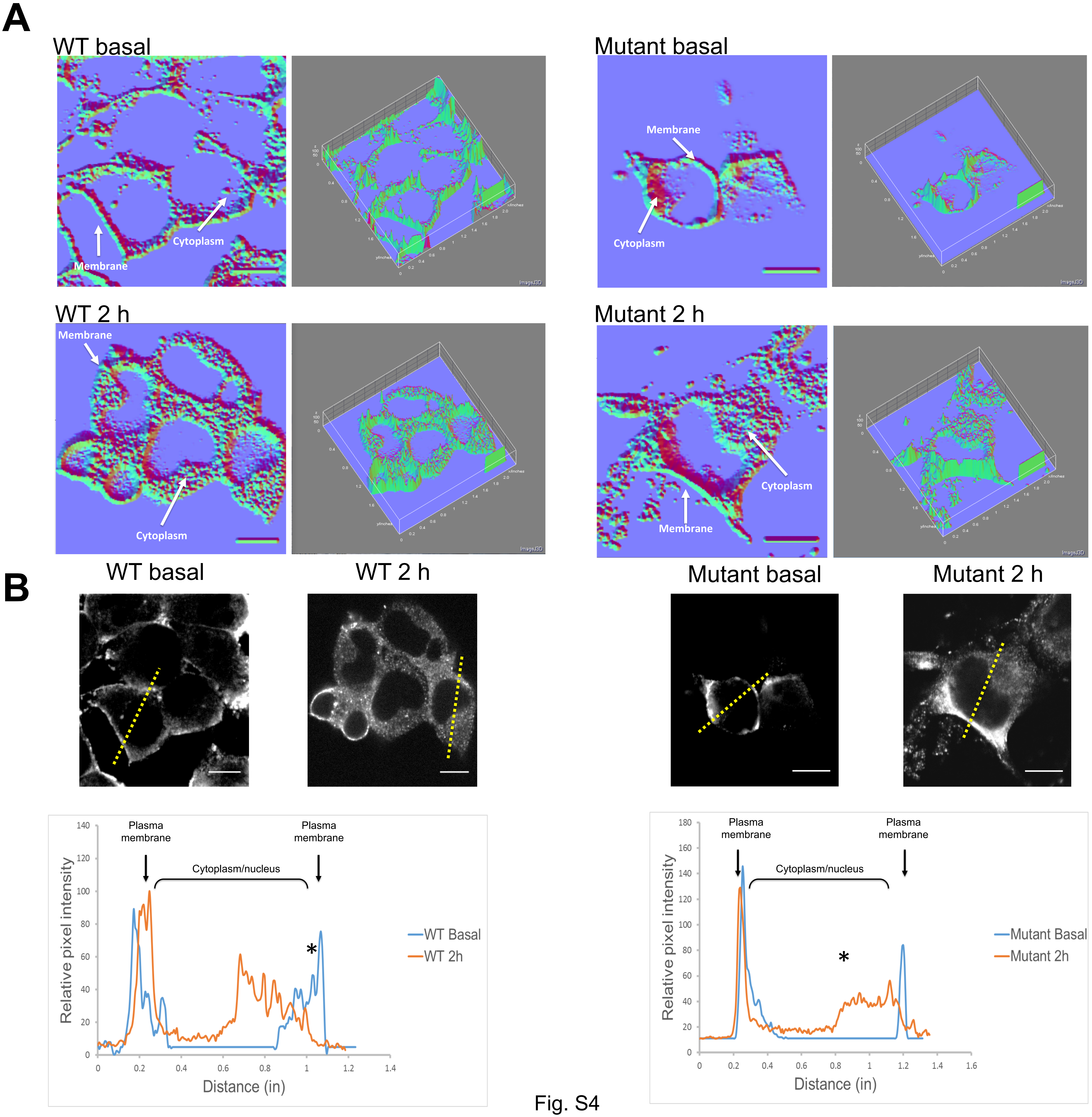
A
WT basal
Membrane
Cytoplasm
Mutant basal
Membrane
Cytoplasm
WT 2 h
Membrane
Cytoplasm
Mutant 2 h
Cytoplasm
Membrane
B
WT basal
WT 2 h
Mutant basal
Mutant 2 h
Plasma membrane
Plasma membrane
Cytoplasm/nucleus
Relative pixel intensity
Distance (in)
WT Basal
WT 2h
*
Plasma membrane
Plasma membrane
Cytoplasm/nucleus
Relative pixel intensity
Distance (in)
Mutant Basal
Mutant 2h
*

Fig. S4

Figure S4. Confocal microscopy analysis of the internalization of WT and mutant FSHR before (basal) and after 2-hours exposure to recombinant FSH. **A:** Spectral pixel-density analysis of the images showed in Fig. 3B. The left and right panels show the distribution of the WT and triple mutant FSHR (green-red clusters over a light blue background), before and after 2 h exposure of transfected HEK-293 cells to FSH. The relocalization of the FSHR to the cytoplasm (white arrows in each spectral representation) after FSH stimulation is evident in both WT and triple mutant FSHR-transfected cells. Each panel also shows a three-dimensional reconstruction of the same images indicating the pixel intensity of the fluorescence given by the FSHRs in each condition (grey background). The visible peaks indicate a higher presence (abundance) of the FSHR variant at a given position. Using these representations, it is possible to observe similar patterns of FSHR re-distribution from the plasma membrane to the cytoplasm upon FSH exposure. **B:** Distribution profiles of WT and mutant FSHR were determined using the confocal images showed in the upper panels. The yellow-dotted line represents the linear selection (corresponding to the distance or X-axis in the graphs) used to plot the corresponding profiles (lower panels), where pixel intensity (fluorescent signal) were calculated and expressed in relative units to build the corresponding graphs. The plots indicate the plasma membrane sections and the higher FSHR peaks (more evident in basal conditions) as lines were traced accross the whole length of a given cell. When cells were stimulated with agonist, FSHR distribution changed and the plots now indicate accumulation (by internalization) of the receptor molecules in the cytoplasm (indicated by the asterisks). Both WT and mutant FSHR showed the same internalization pattern. All analyses were performed with the ImageJ Software (National Institutes of Health, Bethesda, MD, USA).
